# *catena*-Poly[[[bis­[μ-3-(4-carb­oxy­phen­oxy)propionato]-κ^3^ *O* ^1^,*O* ^1′^:*O* ^1^;κ^3^ *O* ^1^:*O* ^1^,*O* ^1′^-bis­[aqua­(*N*,*N*-dimethyl­formamide-κ*O*)cadmium]]-μ-4,4′-bipyridine-κ^2^ *N*:*N*′] dinitrate]

**DOI:** 10.1107/S1600536812002991

**Published:** 2012-01-31

**Authors:** Shan Gao, Seik Weng Ng

**Affiliations:** a Key Laboratory of Functional Inorganic Materials Chemistry, Ministry of Education, Heilongjiang University, Harbin 150080, People’s Republic of China; b Department of Chemistry, University of Malaya, 50603 Kuala Lumpur, Malaysia; c Chemistry Department, Faculty of Science, King Abdulaziz University, PO Box 80203 Jeddah, Saudi Arabia

## Abstract

In the title coordination polymer, {[Cd_2_(C_10_H_9_O_5_)_2_(C_10_H_8_N_2_)_2_(C_3_H_7_NO)_2_(H_2_O)_2_](NO_3_)_2_}_*n*_, the 3-(4-carb­oxy­phen­oxy)propionate monoanion *O*,*O*′-chelates to a Cd^II^ cation through the aliphatic carboxyl­ate end. One of these O atoms is also connected to the metal cation from an inversion-related metal atom. The five O atoms bonded to the metal centre form a penta­gon, above and below which are located the N atoms of the 4,4′-bipyridine mol­ecules. The polycationic ribbon propagates along the *b* axis of the unit cell. The (aromatic) carboxyl end of the monoanion connects adjacent ribbons into a layer motif in the (102) plane. The nitrate ions are hydrogen bonded to the layer. The geometry of the Cd^II^ atom is a *trans*-N_2_O_5_Cd penta­gonal bipyramid.

## Related literature

For 3-(4-carb­oxy­phen­oxy)propionic acid, see: Gao & Ng (2006 ▶).
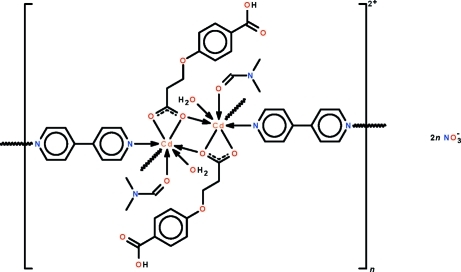


## Experimental

### 

#### Crystal data

[Cd_2_(C_10_H_9_O_5_)_2_(C_10_H_8_N_2_)_2_(C_3_H_7_NO)_2_(H_2_O)_2_](NO_3_)_2_
*M* *_r_* = 1261.76
Triclinic, 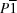

*a* = 9.1020 (5) Å
*b* = 11.6866 (5) Å
*c* = 13.3534 (5) Å
α = 69.1646 (11)°
β = 84.2052 (16)°
γ = 76.9358 (16)°
*V* = 1292.84 (10) Å^3^
*Z* = 1
Mo *K*α radiation
μ = 0.91 mm^−1^
*T* = 293 K
0.19 × 0.12 × 0.11 mm

#### Data collection

Rigaku R-AXIS RAPID IP diffractometer
Absorption correction: multi-scan (*ABSCOR*; Higashi, 1995 ▶) *T*_min_ = 0.847, *T*_max_ = 0.907
12594 measured reflections
5822 independent reflections
4760 reflections with *I* > 2σ(*I*)
*R*_int_ = 0.031

#### Refinement

*R*[*F* ^2^ > 2σ(*F* ^2^)] = 0.030
*wR*(*F* ^2^) = 0.077
*S* = 1.08
5822 reflections
345 parameters
H-atom parameters constrained
Δρ_max_ = 0.95 e Å^−3^
Δρ_min_ = −0.59 e Å^−3^

### 

Data collection: *RAPID-AUTO* (Rigaku, 1998 ▶); cell refinement: *RAPID-AUTO*; data reduction: *CrystalClear* (Rigaku, 2002 ▶); program(s) used to solve structure: *SHELXS97* (Sheldrick, 2008 ▶); program(s) used to refine structure: *SHELXL97* (Sheldrick, 2008 ▶); molecular graphics: *X-SEED* (Barbour, 2001 ▶); software used to prepare material for publication: *publCIF* (Westrip, 2010 ▶).

## Supplementary Material

Crystal structure: contains datablock(s) global, I. DOI: 10.1107/S1600536812002991/bt5796sup1.cif

Structure factors: contains datablock(s) I. DOI: 10.1107/S1600536812002991/bt5796Isup2.hkl

Additional supplementary materials:  crystallographic information; 3D view; checkCIF report

## Figures and Tables

**Table 1:** Hydrogen-bond geometry (Å, °)

*D*—H⋯*A*	*D*—H	H⋯*A*	*D*⋯*A*	*D*—H⋯*A*
O5—H5⋯O4^i^	0.84	1.80	2.629 (3)	171
O1*W*—H11⋯O7	0.84	1.94	2.754 (4)	164
O1*W*—H12⋯O2^ii^	0.84	2.03	2.763 (3)	145

Symmetry codes: (i) 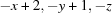; (ii) 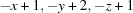.

**Fig. 1.**
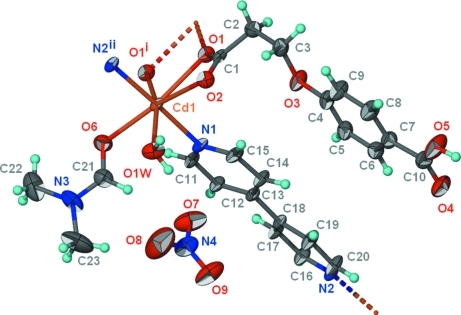
Anitoropic displacement ellipsoid plot (Barbour, 2001) of a portion of polymeric [Cd2(H2O)2(C10H8N2)2(C3H7NO)2(C10H8O5)2]n 2n(NO3) at the 50% probability level; hydrogen atoms are drawn as spheres of arbitrary radius.

**Fig. 2.**
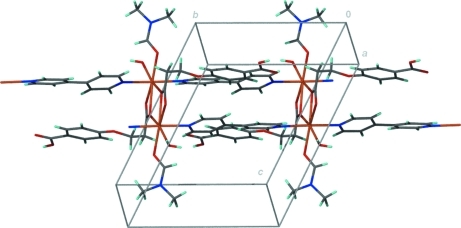
Polycationic ribbon motif.

## supplementary crystallographic information

### Comment

We reported the crystal structure of 3-(4-carboxyphenoxy)propropionic acid (Gao & Ng, 2006). We also reported the crystal structures of some metal derivatives. In the coordination polymer, [Cd_2_(H_2_O)_2_(C_10_H_8_N_2_)_2_(DMF)_2_(C_10_H_8_O_5_)_2_]*_n_* 2*n*(NO_3_) (Scheme I), the 3-(4-carboxyphenoxy)propionate monoanion *O*,*O*'-chelates to a Cd^II^ atom atom through the aliphatic (negatively-charged) carboxyl –CO_2_ end; one of the O atoms is also connected to an inversion-related metal atom. The carboxyl O atoms, the carboxyl O atom of an inversion-related dianion, and the O atoms of the water and DMF molecules comprise a pentagon, above and below which are located the N atoms of the 4,4'-bipyridine heterocycle (Fig. 1). The polycationic ribbon propogates along the *b*-axis of the unit cell; the (aromatic) carboxylic acid end of the monoanion connects adjacent ribbons (Fig. 2) into a layer motif; the nitrate ions are hydrogen bonded to the layer (Table 1).

### Experimental

Cadmium nitrate and 3-(4-carboxyphenoxy)propionic acid (1 mmol) were mixed in a water-DMF (3/1) mixture (10 ml). 4,4'-Bipyridine dissolved in methanol (5 ml) was added. The mixture was stirred until the reactants dissolved. Yellow crystals were isolated after a few days.

### Refinement

Carbon-bound H-atoms were placed in calculated positions (C–H 0.93–0.97 Å) and were included in the refinement in the riding model approximation, with *U*(H) set to 1.2–1.5*U*(C). The acid and water H-atoms were similarly treated (O–H 0.84 Å) and their displacement parameters were similarly tied.

Omitted owing to bad disagreement, the (0 2 0), (10 2 8), (9 2 11), (4 0 13), (4 - 1 14), (1 - 1 14), (10 4 9), (3 - 2 13), (2 - 2 13), (9 3 11), (10 2 9) and (10 5 10) reflections were omitted from refinement.

### Crystal data

**Table:**

[Cd_2_(C_10_H_9_O_5_)_2_(C_10_H_8_N_2_)_2_(C_3_H_7_NO)_2_(H_2_O)_2_](NO_3_)_2_	*Z* = 1
*M**_r_* = 1261.76	*F*(000) = 640
Triclinic, *P*1	*D*_x_ = 1.621 Mg m^−^^3^
Hall symbol: -P 1	Mo *K*α radiation, λ = 0.71073 Å
*a* = 9.1020 (5) Å	Cell parameters from 10397 reflections
*b* = 11.6866 (5) Å	θ = 3.2–27.5°
*c* = 13.3534 (5) Å	µ = 0.91 mm^−^^1^
α = 69.1646 (11)°	*T* = 293 K
β = 84.2052 (16)°	Prism, yellow
γ = 76.9358 (16)°	0.19 × 0.12 × 0.11 mm
*V* = 1292.84 (10) Å^3^	

### Data collection

**Table:**

Rigaku R-AXIS RAPID IP diffractometer	5822 independent reflections
Radiation source: fine-focus sealed tube	4760 reflections with *I* > 2σ(*I*)
graphite	*R*_int_ = 0.031
ω scan	θ_max_ = 27.5°, θ_min_ = 3.2°
Absorption correction: multi-scan (*ABSCOR*; Higashi, 1995)	*h* = −11→11
*T*_min_ = 0.847, *T*_max_ = 0.907	*k* = −15→14
12594 measured reflections	*l* = −17→16

### Refinement

**Table:**

Refinement on *F*^2^	Primary atom site location: structure-invariant direct methods
Least-squares matrix: full	Secondary atom site location: difference Fourier map
*R*[*F*^2^ > 2σ(*F*^2^)] = 0.030	Hydrogen site location: inferred from neighbouring sites
*wR*(*F*^2^) = 0.077	H-atom parameters constrained
*S* = 1.08	*w* = 1/[σ^2^(*F*_o_^2^) + (0.0367*P*)^2^ + 0.2058*P*] where *P* = (*F*_o_^2^ + 2*F*_c_^2^)/3
5822 reflections	(Δ/σ)_max_ = 0.001
345 parameters	Δρ_max_ = 0.95 e Å^−^^3^
0 restraints	Δρ_min_ = −0.59 e Å^−^^3^

### Fractional atomic coordinates and isotropic or equivalent isotropic displacement parameters (Å^2^)

**Table:**

	*x*	*y*	*z*	*U*_iso_*/*U*_eq_	
Cd1	0.18002 (2)	0.944005 (16)	0.589093 (15)	0.02562 (7)	
O1	0.0904 (2)	1.01688 (18)	0.40717 (15)	0.0361 (4)	
O2	0.3311 (2)	1.00556 (19)	0.41763 (15)	0.0390 (5)	
O3	0.3901 (3)	0.8936 (2)	0.23010 (18)	0.0493 (6)	
O4	0.8546 (3)	0.4867 (2)	0.0878 (2)	0.0620 (7)	
O5	0.9522 (3)	0.6549 (2)	0.0014 (2)	0.0741 (8)	
H5	1.0193	0.6153	−0.0288	0.111*	
O6	0.1426 (3)	0.8763 (2)	0.77857 (16)	0.0479 (5)	
O7	0.5403 (4)	0.6153 (3)	0.6982 (3)	0.0893 (10)	
O8	0.7250 (5)	0.6396 (4)	0.7677 (4)	0.1299 (16)	
O9	0.7435 (3)	0.4764 (3)	0.7307 (3)	0.0829 (9)	
O1W	0.4333 (2)	0.8691 (2)	0.64542 (17)	0.0486 (5)	
H11	0.4611	0.7941	0.6493	0.073*	
H12	0.4890	0.9124	0.6010	0.073*	
N1	0.1779 (3)	0.74731 (19)	0.58879 (18)	0.0307 (5)	
N2	0.1773 (3)	0.1380 (2)	0.59570 (18)	0.0315 (5)	
N3	0.2606 (4)	0.7602 (3)	0.9348 (2)	0.0646 (9)	
N4	0.6706 (4)	0.5765 (3)	0.7332 (3)	0.0596 (8)	
C1	0.2171 (3)	1.0305 (2)	0.3627 (2)	0.0282 (5)	
C2	0.2299 (4)	1.0791 (3)	0.2418 (2)	0.0423 (7)	
H2A	0.2158	1.1695	0.2170	0.051*	
H2B	0.1497	1.0586	0.2130	0.051*	
C3	0.3801 (4)	1.0261 (3)	0.1987 (2)	0.0440 (7)	
H3A	0.3853	1.0634	0.1213	0.053*	
H3B	0.4624	1.0431	0.2284	0.053*	
C4	0.5115 (4)	0.8267 (3)	0.1927 (2)	0.0413 (7)	
C5	0.5131 (4)	0.7005 (3)	0.2196 (2)	0.0426 (7)	
H5A	0.4373	0.6657	0.2639	0.051*	
C6	0.6268 (4)	0.6263 (3)	0.1807 (2)	0.0412 (7)	
H6	0.6275	0.5416	0.1990	0.049*	
C7	0.7403 (4)	0.6773 (3)	0.1143 (2)	0.0415 (7)	
C8	0.7405 (4)	0.8018 (3)	0.0913 (3)	0.0515 (8)	
H8	0.8178	0.8360	0.0485	0.062*	
C9	0.6276 (4)	0.8772 (3)	0.1307 (3)	0.0514 (8)	
H9	0.6299	0.9608	0.1156	0.062*	
C10	0.8552 (4)	0.5984 (3)	0.0665 (3)	0.0481 (8)	
C11	0.0810 (3)	0.6805 (2)	0.6513 (2)	0.0339 (6)	
H11A	0.0148	0.7150	0.6957	0.041*	
C12	0.0742 (3)	0.5627 (2)	0.6532 (2)	0.0330 (6)	
H12A	0.0044	0.5198	0.6978	0.040*	
C13	0.1722 (3)	0.5090 (2)	0.5881 (2)	0.0279 (5)	
C14	0.2714 (3)	0.5799 (2)	0.5215 (2)	0.0366 (6)	
H14	0.3376	0.5486	0.4752	0.044*	
C15	0.2709 (3)	0.6963 (3)	0.5248 (2)	0.0362 (6)	
H15	0.3385	0.7419	0.4802	0.043*	
C16	0.0616 (3)	0.2007 (3)	0.6371 (2)	0.0376 (7)	
H16	−0.0188	0.1622	0.6681	0.045*	
C17	0.0552 (3)	0.3203 (3)	0.6364 (2)	0.0381 (7)	
H17	−0.0282	0.3605	0.6661	0.046*	
C18	0.1735 (3)	0.3800 (2)	0.5914 (2)	0.0278 (5)	
C19	0.2945 (4)	0.3138 (3)	0.5491 (3)	0.0428 (7)	
H19	0.3768	0.3498	0.5180	0.051*	
C20	0.2921 (4)	0.1946 (3)	0.5536 (3)	0.0448 (8)	
H20	0.3748	0.1515	0.5258	0.054*	
C21	0.2327 (4)	0.7857 (3)	0.8328 (3)	0.0483 (8)	
H21	0.2858	0.7304	0.7998	0.058*	
C22	0.1831 (6)	0.8376 (5)	0.9937 (4)	0.0898 (16)	
H22A	0.1137	0.9066	0.9477	0.135*	
H22B	0.2548	0.8687	1.0197	0.135*	
H22C	0.1286	0.7895	1.0532	0.135*	
C23	0.3709 (7)	0.6488 (6)	0.9891 (4)	0.118 (2)	
H23A	0.4105	0.6049	0.9405	0.177*	
H23B	0.3234	0.5955	1.0496	0.177*	
H23C	0.4516	0.6725	1.0131	0.177*	

### Atomic displacement parameters (Å^2^)

**Table:**

	*U*^11^	*U*^22^	*U*^33^	*U*^12^	*U*^13^	*U*^23^
Cd1	0.02636 (11)	0.01654 (10)	0.03726 (12)	−0.00540 (7)	−0.00062 (7)	−0.01261 (7)
O1	0.0260 (11)	0.0386 (11)	0.0424 (11)	−0.0067 (9)	0.0057 (8)	−0.0140 (9)
O2	0.0319 (12)	0.0424 (12)	0.0433 (11)	−0.0115 (9)	0.0017 (9)	−0.0139 (9)
O3	0.0467 (14)	0.0443 (13)	0.0622 (14)	−0.0143 (11)	0.0238 (11)	−0.0283 (11)
O4	0.0743 (19)	0.0414 (14)	0.0691 (16)	−0.0119 (13)	0.0278 (13)	−0.0256 (12)
O5	0.071 (2)	0.0525 (16)	0.101 (2)	−0.0199 (14)	0.0489 (16)	−0.0380 (14)
O6	0.0440 (14)	0.0504 (14)	0.0415 (12)	−0.0020 (11)	−0.0028 (10)	−0.0107 (10)
O7	0.053 (2)	0.0577 (19)	0.136 (3)	−0.0091 (15)	−0.0075 (18)	−0.0079 (17)
O8	0.117 (3)	0.103 (3)	0.207 (5)	−0.016 (3)	−0.031 (3)	−0.094 (3)
O9	0.0556 (19)	0.0490 (17)	0.140 (3)	−0.0094 (14)	0.0235 (17)	−0.0346 (17)
O1W	0.0314 (12)	0.0417 (13)	0.0651 (14)	−0.0108 (10)	−0.0036 (10)	−0.0068 (10)
N1	0.0271 (13)	0.0192 (11)	0.0485 (13)	−0.0045 (9)	0.0000 (10)	−0.0154 (9)
N2	0.0354 (14)	0.0206 (11)	0.0442 (13)	−0.0074 (10)	0.0013 (10)	−0.0176 (9)
N3	0.067 (2)	0.085 (2)	0.0376 (16)	−0.0242 (19)	−0.0111 (14)	−0.0075 (15)
N4	0.048 (2)	0.0435 (18)	0.082 (2)	−0.0156 (15)	0.0143 (16)	−0.0162 (16)
C1	0.0261 (15)	0.0209 (13)	0.0382 (14)	−0.0021 (10)	0.0038 (11)	−0.0136 (10)
C2	0.0413 (19)	0.0419 (18)	0.0391 (16)	−0.0022 (14)	0.0030 (13)	−0.0133 (13)
C3	0.045 (2)	0.0462 (19)	0.0393 (16)	−0.0087 (15)	0.0126 (13)	−0.0168 (13)
C4	0.0386 (18)	0.0471 (19)	0.0437 (16)	−0.0108 (14)	0.0100 (13)	−0.0241 (14)
C5	0.046 (2)	0.0458 (19)	0.0416 (16)	−0.0171 (15)	0.0112 (13)	−0.0201 (14)
C6	0.0432 (19)	0.0402 (17)	0.0424 (16)	−0.0103 (14)	0.0013 (13)	−0.0165 (13)
C7	0.0402 (19)	0.0414 (18)	0.0432 (16)	−0.0053 (14)	0.0039 (13)	−0.0178 (13)
C8	0.043 (2)	0.048 (2)	0.064 (2)	−0.0125 (16)	0.0207 (16)	−0.0238 (16)
C9	0.053 (2)	0.0385 (18)	0.064 (2)	−0.0141 (16)	0.0206 (16)	−0.0226 (15)
C10	0.052 (2)	0.044 (2)	0.0466 (18)	−0.0062 (16)	0.0070 (15)	−0.0173 (14)
C11	0.0347 (16)	0.0250 (14)	0.0473 (16)	−0.0079 (12)	0.0073 (12)	−0.0198 (12)
C12	0.0342 (16)	0.0243 (14)	0.0431 (15)	−0.0092 (12)	0.0067 (12)	−0.0146 (11)
C13	0.0296 (15)	0.0186 (13)	0.0395 (14)	−0.0058 (10)	−0.0031 (11)	−0.0135 (10)
C14	0.0381 (17)	0.0232 (14)	0.0516 (17)	−0.0076 (12)	0.0113 (13)	−0.0192 (12)
C15	0.0321 (16)	0.0223 (14)	0.0550 (17)	−0.0101 (12)	0.0089 (13)	−0.0142 (12)
C16	0.0368 (17)	0.0273 (15)	0.0560 (18)	−0.0141 (13)	0.0092 (13)	−0.0212 (13)
C17	0.0345 (17)	0.0276 (15)	0.0587 (18)	−0.0085 (12)	0.0114 (13)	−0.0247 (13)
C18	0.0285 (15)	0.0208 (13)	0.0392 (14)	−0.0050 (10)	−0.0014 (11)	−0.0161 (11)
C19	0.0383 (18)	0.0313 (16)	0.069 (2)	−0.0170 (13)	0.0179 (15)	−0.0288 (14)
C20	0.0414 (19)	0.0325 (16)	0.072 (2)	−0.0119 (14)	0.0160 (15)	−0.0332 (15)
C21	0.045 (2)	0.052 (2)	0.0461 (18)	−0.0156 (16)	0.0003 (15)	−0.0119 (15)
C22	0.113 (4)	0.113 (4)	0.062 (3)	−0.055 (3)	0.011 (3)	−0.037 (3)
C23	0.119 (5)	0.124 (5)	0.078 (3)	−0.006 (4)	−0.051 (3)	0.007 (3)

### Geometric parameters (Å, °)

**Table:**

Cd1—O1	2.4304 (19)	C4—C9	1.380 (4)
Cd1—O1^i^	2.398 (2)	C4—C5	1.386 (4)
Cd1—O2	2.5080 (19)	C5—C6	1.378 (4)
Cd1—O6	2.381 (2)	C5—H5A	0.9300
Cd1—O1W	2.370 (2)	C6—C7	1.388 (4)
Cd1—N1	2.305 (2)	C6—H6	0.9300
Cd1—N2^ii^	2.294 (2)	C7—C8	1.376 (4)
O1—C1	1.259 (3)	C7—C10	1.482 (4)
O1—Cd1^i^	2.3985 (19)	C8—C9	1.387 (4)
O2—C1	1.251 (3)	C8—H8	0.9300
O3—C4	1.363 (4)	C9—H9	0.9300
O3—C3	1.436 (4)	C11—C12	1.384 (3)
O4—C10	1.235 (4)	C11—H11A	0.9300
O5—C10	1.293 (4)	C12—C13	1.387 (4)
O5—H5	0.8400	C12—H12A	0.9300
O6—C21	1.225 (4)	C13—C14	1.394 (4)
O7—N4	1.245 (4)	C13—C18	1.490 (3)
O8—N4	1.206 (4)	C14—C15	1.376 (4)
O9—N4	1.218 (4)	C14—H14	0.9300
O1W—H11	0.8400	C15—H15	0.9300
O1W—H12	0.8400	C16—C17	1.382 (4)
N1—C11	1.335 (4)	C16—H16	0.9300
N1—C15	1.338 (3)	C17—C18	1.385 (4)
N2—C20	1.332 (4)	C17—H17	0.9300
N2—C16	1.333 (4)	C18—C19	1.389 (4)
N2—Cd1^iii^	2.294 (2)	C19—C20	1.379 (4)
N3—C21	1.324 (4)	C19—H19	0.9300
N3—C22	1.428 (5)	C20—H20	0.9300
N3—C23	1.453 (6)	C21—H21	0.9300
C1—C2	1.510 (4)	C22—H22A	0.9600
C2—C3	1.515 (4)	C22—H22B	0.9600
C2—H2A	0.9700	C22—H22C	0.9600
C2—H2B	0.9700	C23—H23A	0.9600
C3—H3A	0.9700	C23—H23B	0.9600
C3—H3B	0.9700	C23—H23C	0.9600
			
N2^ii^—Cd1—N1	177.79 (8)	C6—C5—H5A	120.0
N2^ii^—Cd1—O1W	92.00 (8)	C4—C5—H5A	120.0
N1—Cd1—O1W	88.38 (8)	C5—C6—C7	120.4 (3)
N2^ii^—Cd1—O6	87.14 (8)	C5—C6—H6	119.8
N1—Cd1—O6	90.79 (8)	C7—C6—H6	119.8
O1W—Cd1—O6	79.50 (8)	C8—C7—C6	119.0 (3)
N2^ii^—Cd1—O1^i^	91.36 (8)	C8—C7—C10	121.4 (3)
N1—Cd1—O1^i^	87.59 (7)	C6—C7—C10	119.7 (3)
O1W—Cd1—O1^i^	161.18 (7)	C7—C8—C9	121.2 (3)
O6—Cd1—O1^i^	82.19 (7)	C7—C8—H8	119.4
N2^ii^—Cd1—O1	96.38 (7)	C9—C8—H8	119.4
N1—Cd1—O1	85.10 (7)	C4—C9—C8	119.2 (3)
O1W—Cd1—O1	127.31 (7)	C4—C9—H9	120.4
O6—Cd1—O1	152.60 (8)	C8—C9—H9	120.4
O1^i^—Cd1—O1	70.59 (7)	O4—C10—O5	123.4 (3)
N2^ii^—Cd1—O2	87.11 (7)	O4—C10—C7	121.0 (3)
N1—Cd1—O2	95.10 (7)	O5—C10—C7	115.6 (3)
O1W—Cd1—O2	76.40 (7)	N1—C11—C12	123.3 (3)
O6—Cd1—O2	154.98 (8)	N1—C11—H11A	118.4
O1^i^—Cd1—O2	122.27 (6)	C12—C11—H11A	118.4
O1—Cd1—O2	52.37 (6)	C11—C12—C13	119.5 (3)
C1—O1—Cd1^i^	153.61 (18)	C11—C12—H12A	120.3
C1—O1—Cd1	95.24 (16)	C13—C12—H12A	120.3
Cd1^i^—O1—Cd1	109.41 (7)	C12—C13—C14	117.1 (2)
C1—O2—Cd1	91.80 (16)	C12—C13—C18	121.2 (2)
C4—O3—C3	117.4 (2)	C14—C13—C18	121.7 (2)
C10—O5—H5	120.0	C15—C14—C13	119.6 (3)
C21—O6—Cd1	117.7 (2)	C15—C14—H14	120.2
Cd1—O1W—H11	109.5	C13—C14—H14	120.2
Cd1—O1W—H12	109.5	N1—C15—C14	123.3 (3)
H11—O1W—H12	109.5	N1—C15—H15	118.4
C11—N1—C15	117.2 (2)	C14—C15—H15	118.4
C11—N1—Cd1	121.05 (17)	N2—C16—C17	123.2 (3)
C15—N1—Cd1	121.72 (18)	N2—C16—H16	118.4
C20—N2—C16	117.1 (2)	C17—C16—H16	118.4
C20—N2—Cd1^iii^	120.16 (18)	C16—C17—C18	119.8 (3)
C16—N2—Cd1^iii^	122.71 (18)	C16—C17—H17	120.1
C21—N3—C22	121.9 (4)	C18—C17—H17	120.1
C21—N3—C23	119.2 (4)	C17—C18—C19	116.8 (2)
C22—N3—C23	119.0 (4)	C17—C18—C13	122.0 (2)
O8—N4—O9	120.3 (4)	C19—C18—C13	121.2 (2)
O8—N4—O7	119.6 (4)	C20—C19—C18	119.7 (3)
O9—N4—O7	120.1 (4)	C20—C19—H19	120.2
O2—C1—O1	120.6 (2)	C18—C19—H19	120.2
O2—C1—C2	120.1 (2)	N2—C20—C19	123.4 (3)
O1—C1—C2	119.3 (2)	N2—C20—H20	118.3
C1—C2—C3	113.3 (3)	C19—C20—H20	118.3
C1—C2—H2A	108.9	O6—C21—N3	125.3 (4)
C3—C2—H2A	108.9	O6—C21—H21	117.4
C1—C2—H2B	108.9	N3—C21—H21	117.4
C3—C2—H2B	108.9	N3—C22—H22A	109.5
H2A—C2—H2B	107.7	N3—C22—H22B	109.5
O3—C3—C2	106.8 (2)	H22A—C22—H22B	109.5
O3—C3—H3A	110.4	N3—C22—H22C	109.5
C2—C3—H3A	110.4	H22A—C22—H22C	109.5
O3—C3—H3B	110.4	H22B—C22—H22C	109.5
C2—C3—H3B	110.4	N3—C23—H23A	109.5
H3A—C3—H3B	108.6	N3—C23—H23B	109.5
O3—C4—C9	124.3 (3)	H23A—C23—H23B	109.5
O3—C4—C5	115.8 (3)	N3—C23—H23C	109.5
C9—C4—C5	120.0 (3)	H23A—C23—H23C	109.5
C6—C5—C4	120.1 (3)	H23B—C23—H23C	109.5
			
N2^ii^—Cd1—O1—C1	81.21 (16)	C3—O3—C4—C9	2.8 (5)
N1—Cd1—O1—C1	−100.43 (16)	C3—O3—C4—C5	−176.5 (3)
O1W—Cd1—O1—C1	−16.23 (19)	O3—C4—C5—C6	176.5 (3)
O6—Cd1—O1—C1	177.34 (16)	C9—C4—C5—C6	−2.8 (5)
O1^i^—Cd1—O1—C1	170.41 (19)	C4—C5—C6—C7	−0.1 (5)
O2—Cd1—O1—C1	−0.14 (14)	C5—C6—C7—C8	2.3 (5)
N2^ii^—Cd1—O1—Cd1^i^	−89.19 (9)	C5—C6—C7—C10	−175.3 (3)
N1—Cd1—O1—Cd1^i^	89.16 (9)	C6—C7—C8—C9	−1.7 (5)
O1W—Cd1—O1—Cd1^i^	173.37 (7)	C10—C7—C8—C9	175.9 (3)
O6—Cd1—O1—Cd1^i^	6.93 (19)	O3—C4—C9—C8	−175.8 (3)
O1^i^—Cd1—O1—Cd1^i^	0.0	C5—C4—C9—C8	3.4 (5)
O2—Cd1—O1—Cd1^i^	−170.55 (12)	C7—C8—C9—C4	−1.2 (6)
N2^ii^—Cd1—O2—C1	−100.20 (16)	C8—C7—C10—O4	179.8 (3)
N1—Cd1—O2—C1	79.95 (16)	C6—C7—C10—O4	−2.6 (5)
O1W—Cd1—O2—C1	167.04 (16)	C8—C7—C10—O5	−1.0 (5)
O6—Cd1—O2—C1	−177.12 (17)	C6—C7—C10—O5	176.6 (3)
O1^i^—Cd1—O2—C1	−10.41 (17)	C15—N1—C11—C12	0.7 (4)
O1—Cd1—O2—C1	0.14 (14)	Cd1—N1—C11—C12	179.3 (2)
N2^ii^—Cd1—O6—C21	−125.3 (2)	N1—C11—C12—C13	0.3 (4)
N1—Cd1—O6—C21	55.5 (2)	C11—C12—C13—C14	−1.4 (4)
O1W—Cd1—O6—C21	−32.7 (2)	C11—C12—C13—C18	177.1 (3)
O1^i^—Cd1—O6—C21	142.9 (2)	C12—C13—C14—C15	1.5 (4)
O1—Cd1—O6—C21	136.3 (2)	C18—C13—C14—C15	−177.0 (3)
O2—Cd1—O6—C21	−48.4 (3)	C11—N1—C15—C14	−0.6 (4)
O1W—Cd1—N1—C11	119.7 (2)	Cd1—N1—C15—C14	−179.2 (2)
O6—Cd1—N1—C11	40.2 (2)	C13—C14—C15—N1	−0.5 (5)
O1^i^—Cd1—N1—C11	−41.9 (2)	C20—N2—C16—C17	1.1 (5)
O1—Cd1—N1—C11	−112.6 (2)	Cd1^iii^—N2—C16—C17	−176.5 (2)
O2—Cd1—N1—C11	−164.1 (2)	N2—C16—C17—C18	−0.3 (5)
O1W—Cd1—N1—C15	−61.8 (2)	C16—C17—C18—C19	−0.3 (4)
O6—Cd1—N1—C15	−141.2 (2)	C16—C17—C18—C13	179.7 (3)
O1^i^—Cd1—N1—C15	136.6 (2)	C12—C13—C18—C17	17.2 (4)
O1—Cd1—N1—C15	65.9 (2)	C14—C13—C18—C17	−164.4 (3)
O2—Cd1—N1—C15	14.4 (2)	C12—C13—C18—C19	−162.9 (3)
Cd1—O2—C1—O1	−0.3 (2)	C14—C13—C18—C19	15.6 (4)
Cd1—O2—C1—C2	178.9 (2)	C17—C18—C19—C20	0.0 (5)
Cd1^i^—O1—C1—O2	159.6 (3)	C13—C18—C19—C20	180.0 (3)
Cd1—O1—C1—O2	0.3 (3)	C16—N2—C20—C19	−1.4 (5)
Cd1^i^—O1—C1—C2	−19.6 (5)	Cd1^iii^—N2—C20—C19	176.2 (3)
Cd1—O1—C1—C2	−178.9 (2)	C18—C19—C20—N2	0.9 (5)
O2—C1—C2—C3	33.5 (4)	Cd1—O6—C21—N3	156.6 (3)
O1—C1—C2—C3	−147.4 (3)	C22—N3—C21—O6	1.3 (6)
C4—O3—C3—C2	174.9 (3)	C23—N3—C21—O6	179.9 (4)
C1—C2—C3—O3	63.2 (3)		

Symmetry codes: (i) −*x*, −*y*+2, −*z*+1; (ii) *x*, *y*+1, *z*; (iii) *x*, *y*−1, *z*.

### Hydrogen-bond geometry (Å, °)

**Table:**

*D*—H···*A*	*D*—H	H···*A*	*D*···*A*	*D*—H···*A*
O5—H5···O4^iv^	0.84	1.80	2.629 (3)	171
O1W—H11···O7	0.84	1.94	2.754 (4)	164
O1W—H12···O2^v^	0.84	2.03	2.763 (3)	145

Symmetry codes: (iv) −*x*+2, −*y*+1, −*z*; (v) −*x*+1, −*y*+2, −*z*+1.
